# Low genetic diversity in the locus encoding the *Plasmodium vivax* P41 protein in Colombia’s parasite population

**DOI:** 10.1186/1475-2875-13-388

**Published:** 2014-09-30

**Authors:** Johanna Forero-Rodríguez, Diego Garzón-Ospina, Manuel A Patarroyo

**Affiliations:** Fundación Instituto de Inmunología de Colombia (FIDIC), Carrera 50 No. 26-20, Bogotá, DC, Colombia; Microbiology Postgraduate Programme, Universidad Nacional de Colombia, Bogotá, DC, Colombia; School of Medicine and Health Sciences, Universidad del Rosario, Bogotá, DC, Colombia

**Keywords:** *Plasmodium vivax*, 6-Cys, *pv41*, s48/45 domains, Genetic variability, Functional constraint, Anti-malarial vaccine

## Abstract

**Background:**

The development of malaria vaccine has been hindered by the allele-specific responses produced by some parasite antigens’ high genetic diversity. Such antigen genetic diversity must thus be evaluated when designing a completely effective vaccine. *Plasmodium falciparum* P12, P38 and P41 proteins have red blood cell binding regions in the s48/45 domains and are located on merozoite surface, P41 forming a heteroduplex with P12. These three genes have been identified in *Plasmodium vivax* and share similar characteristics with their orthologues in *Plasmodium falciparum. Plasmodium vivax pv12* and *pv38* have low genetic diversity but *pv41* polymorphism has not been described.

**Methods:**

The present study was aimed at evaluating the *P. vivax p41* (*pv41*) gene’s polymorphism. DNA sequences from Colombian clinical isolates from *pv41* gene were analysed for characterising and studying the genetic diversity and the evolutionary forces that produced the variation pattern so observed.

**Results:**

Similarly to other members of the 6-Cys family, *pv41* had low genetic polymorphism. *pv41* 3′-end displayed the highest nucleotide diversity value; several substitutions found there were under positive selection. Negatively selected codons at inter-species level were identified in the s48/45 domains; *p41* would thus seem to have functional/structural constraints due to the presence of these domains.

**Conclusions:**

In spite of the functional constraints of Pv41 s48/45 domains, immune system pressure seems to have allowed non-synonymous substitutions to become fixed within them as an adaptation mechanism; including Pv41 s48/45 domains in a vaccine should thus be carefully evaluated due to these domains containing some allele variants.

**Electronic supplementary material:**

The online version of this article (doi:10.1186/1475-2875-13-388) contains supplementary material, which is available to authorized users.

## Background

Of the five malaria parasites (*Plasmodium falciparum, Plasmodium vivax, Plasmodium malarie, Plasmodium ovale* and *Plasmodium knowlesi*) affecting human beings, *P. falciparum* is the species causing the most severe clinical manifestations, whilst *P. vivax* is the species most widely distributed throughout the world, mainly affecting the Asian and American continents and causing the highest morbidity outside of Africa. In spite of efforts to date for controlling malaria, it continues to be a serious public health problem; 18.9 million cases of *P. vivax* occurred in 2012, children under five years old and pregnant women being the most vulnerable populations [1].

An anti-malarial vaccine represents one of the alternative control measures regarding this disease; developing a multi-antigen vaccine against the parasite’s blood stage is focused on blocking all interactions with a host cell, thereby avoiding recognition and subsequent invasion. Several antigens have been proposed as vaccine candidates [2–4]; however, as many of them have high genetic diversity [5–12], this is an obstacle regarding such proposal [13, 14] since they induce allele-specific immune responses [15]. The genetic diversity of candidate antigens must thus be evaluated [14, 16] for selecting the most frequent variants or conserved domains [13, 14].

Proteins involved in red blood cell (RBC) invasion have been characterized in merozoite surface regions known as detergent-resistant membranes (DRM) [17–19], many of these being potential vaccine candidates [4, 20, 21]. Such DRMs include a group of proteins belonging to the 6-Cys family (P12, P38, P41 and P92) which is characterised by the presence of domains containing six conserved cysteines called s48/45 [17, 22–24]. The *P. falciparum* P41 (Pf41) protein has two high-activity binding peptides in the s48/45 domains [17], thereby suggesting a role in RBC invasion. This protein does not have GPI-anchored domains and its presence on merozoite membrane is due to the formation of an inverted heteroduplex with Pf12 [25, 26]. The *pv41* gene has recently been characterised in *P. vivax* (*pv41*) [22, 27]; this gene encodes a 385 residue-long membrane protein. Similar to its orthologue in *P. falciparum*, the protein has a signal peptide and two s48/45 domains but no GPI-anchor. The *P. vivax* P41 (Pv41) protein has been shown to be antigenic [27, 28], suggesting that it is exposed to the host immune system, probably during invasion of the host cell.

Given that Pv41 has been located on merozoite surface and that it has no membrane anchoring domains [22, 27], it could be interacting with another protein anchored to parasite surface. This protein’s similarity with its orthologue in *P. falciparum* suggests that Pv41 might form a complex with Pv12, a protein which has been shown to be highly conserved [29]. The present study was therefore aimed at using population genetics analysis for evaluating the *pv41* gene’s genetic diversity by determining the evolutionary processes producing the locus’s variation pattern. The results showed that *pv41* had low genetic diversity, the gene’s 3′-end region being the most diverse, fixing mutations by positive selection, probably as a mechanism for evading the immune system. Like other members of the 6-Cys family, this gene seemed to have functional constraints due to the presence of s48/45 domains.

## Methods

### Declaration of ethical considerations

**Figure 1:**
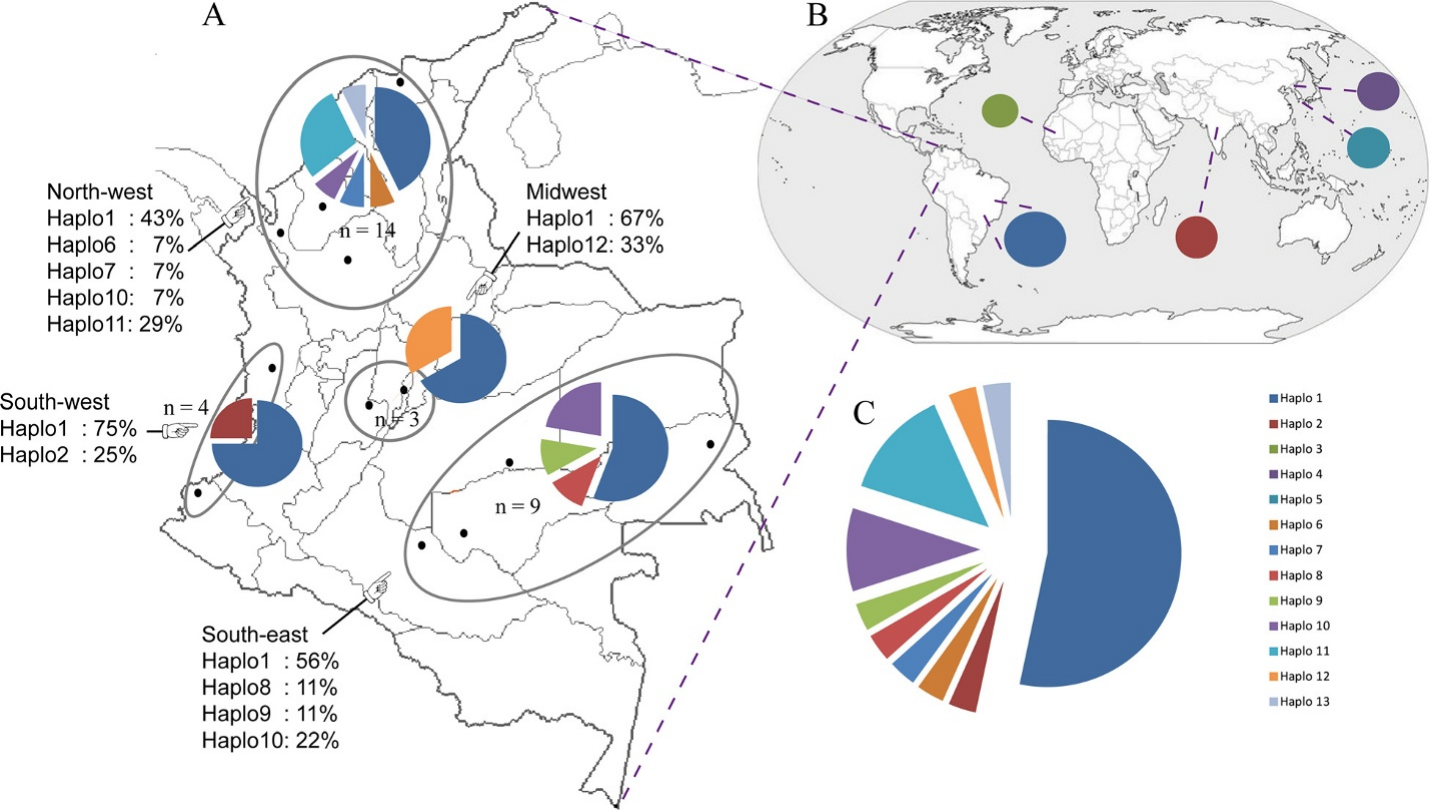
***pv41*** **haplotype distribution in the Colombian population and origin of the reference strain sequences.** Panel **A** shows the haplotype distribution found in *pv41*. Panel **B** shows the origin of the reference strain sequences and panel **C** represents haplotype frequency in the Colombian population. Haplotype 1: Sal-I, Brazil-I, haplotype 2: India, haplotype 3: Mauritania, haplotype 4: North Korea, haplotype 5: South Korea, haplotype 1–2 and 6–13: Colombian isolates.

This study involved using thirty *P. vivax*-infected samples collected between 2007 and 2010 (2007: 5 isolates, 2008: 3 isolates, 2009: 8 isolates, 2010: 14 isolates); they had been obtained from different regions of Colombia (Figure 1, South-west: Chocó, Nariño; South-east: Caquetá, Guainía, Guaviare, Meta; Midwest: Bogotá, Tolima; North-west: Atlántico, Antioquia, Córdoba). All *P. vivax*-infected patients who provided blood samples were notified of the study’s objective and then signed an informed consent form. All the procedures involved in taking the samples had already been approved by the Fundación Instituto de Inmunología de Colombia’s (FIDIC) ethics’ committee.

### Genotyping *Plasmodium vivax*samples

PCR-RFLP of the *pvmsp-1* polymorphic marker was used for identifying/analysing different genotypes in the samples and infection by a single *P. vivax* strain, as described previously [30]. Briefly, this gene’s blocks 6, 7 and 8 were amplified with direct 5′-AAAATCGAGAGCATGATCGCCACTGAGAAG-3′ and reverse 5′-AGCTTGTACTTTCCATAGTGGTCCAG-3′ primers. The amplified fragments were digested with Alu I and Mnl I restriction enzymes.

### PCR amplification of the *pv41*gene

Previously reported primers were used for amplifying *pv41* [22]. The PCR reaction mixture contained 10 mM Tris HCl, 50 mM KCl (GeneAmp 10X PCR Buffer II (Applied Biosystems)), 1.5 mM MgCl_2_, 0.2 mM of each dNTP, 0.5 μM of each primer (direct 5′ ATGAAAAGGCTCCTCCTGC 3′ and reverse 5′ CTCCTGGAAGGACTTGGC 3′), 0.76 U Amplitaq Gold DNA polymerase (Applied Biosystems) and 40 ng genomic DNA at 50 μL final volume. The PCR thermal profile was as follows: one cycle at 95°C (7 min), 40 cycles at 95°C (20 sec), 60°C (30 sec), 72°C (1 min) and a final extension cycle at 72°C (10 min). The amplification products were purified using an UltraClean PCR Clean-up kit (MO BIO). The purified PCR products were bidirectionally sequenced with the amplification primers using the BigDye method with capillary electrophoresis, using the ABI-3730 XL sequencer (MACROGEN, Seoul, South Korea). Two independent PCR products were sequenced per sample to rule out errors.

### Analysing genetic diversity

CLC Main workbench software v.5 (CLC bio, Cambridge, MA, USA) was used for analysing and assembling the electropherograms obtained by sequencing, giving one sequence per sample. The 30 sequences obtained from Colombian isolates were compared to and analysed regarding reference sequences obtained from several sequencing projects [31, 32] (PlasmoDB accession number: PVX_000995, GenBank accession number: AFNI01000110.1, AFNJ01000259.1, AFMK01000149.1 and AFBK01000223.1) or reported in databases (GenBank accession number: GU476495.1). These 36 sequences were then compared to *Plasmodium cynomolgi* (GenBank accession number*:* BAEJ01000104.1) and *P. knowlesi* orthologous sequences (PlasmoDB accession number: PKH_030970), two species which are phylogenetically close to *P. vivax* [33]. Gene Runner software was used for translating all the sequences for obtaining the deduced amino acid sequences; the MUSCLE algorithm was then used for aligning such sequences [34] and then edited manually. The PAL2NAL web-based tool [35] was then used for converting protein alignments into their respective nucleotide alignments.

DnaSP v.5 software [36] was used for quantifying *pv41* genetic polymorphism by calculating: the number of segregant sites (Ss), the number of singleton sites (s), the number of parsimony-informative sites (Ps), the number of haplotypes (H), haplotype diversity (Hd, multiplied by (n-1)/n, according to Depaulis and Veuille [36, 37]), the Watterson estimator (θ^w^), the average number of nucleotide differences (*k*) and nucleotide diversity per site (π). Data was obtained for the reference sequences plus the Colombian sequences (worldwide diversity), as well as for just the Colombian sequences (local diversity).

The Colombian parasite population sequences were used for evaluating the neutral model of molecular evolution using tests based on the frequency spectrum of nucleotide polymorphisms and haplotype distribution. Tajima’s D test [38], Fu and Li’s D* and F* tests [39], and Fay and Wu’s H test [40] were calculated for the first group of tests. Fu’s Fs test [41] and K-test and H-test [37] were calculated as part of the group of tests based on haplotype distribution. The significance of all tests was determined by coalescence simulations using DnaSP v.5 [36] and ALLELIX software (provided by Dr Sylvain Mousset). Sites having gaps were not taken into account for all tests.

The effect of natural selection was evaluated by calculating the difference between the average number of non-synonymous substitutions per non-synonymous site (d_N_) and the average number of synonymous substitutions per synonymous site (d_S_) using the modified Nei-Gojobori method [42]. Significance was determined by using Fisher’s exact tests and the Z test incorporated in MEGA v.5 software [43]. SLAC, FEL, REL [44], IFEL [45], MEME [46] and FUBAR methods [47] were used for calculating the ω (d_N_/d_S_) value for each codon in the *pv41* alignment.

The McDonald-Kreitman test [48] was calculated for evaluating the effect of natural selection on *p41* during the evolutionary history of *P. vivax* and related species (*Plasmodium cynomolgi* and *P. knowlesi*); this test compared intraspecific polymorphism with interspecific divergence using a web server [49], which takes the Jukes-Cantor distance correction regarding divergence per site [50] into account. The Nei-Gojobori modified method [42] was also used for calculating the difference between non-synonymous (K_N_) and synonymous (K_S_) divergence rates using Jukes-Cantor divergence correction [50]. Significant values were determined by using the Z test incorporate in MEGA v.5 software [43]. SLAC, FEL, REL [44], MEME [46] and FUBAR [47] methods were used for determining sites under interspecies selection using the *P. vivax*, *P. cynomolgi* and *P. knowlesi* sequences as data set.

Z_nS_ [51] and ZZ [52] tests were calculated for evaluating non-random associations between polymorphisms (linkage disequilibrium or LD) and the influence of intragenic recombination on *pv41*. The minimum number of recombination events (Rm) [53] was also calculated and the GARD method [54] available from Datamonkey [55] was used for evaluating recombination processes.

## Results

### Genetic diversity in *pv41*

Thirty *P. vivax*-infected samples, obtained from different parts of Colombia (Figure 1), were genotyped using the *pvmsp-1* polymorphic marker. The RFLP patterns produced from *pvmsp-1* blocks 6–8 suggested the presence of different genotypes in the aforementioned samples as well as single strain infections in each sample. Taking into account that all these samples have been previously used in other studies involving genes having high polymorphism [6], in which none of the electropherograms revealed overlapping peaks during the sequencing, we can ascertain the absence of multiple infections.

The 30 genotyped isolates had a 1,152 base pair (bp) fragment corresponding to the *pv41* gene*.* The sequences obtained from these 30 isolates (Additional file 1) were compared to and analysed together with sequences reported by several sequencing projects [31, 32]. Sequences having a different haplotype were deposited in the GenBank database (accession numbers KM212268-KM212275).

**Figure 2:**
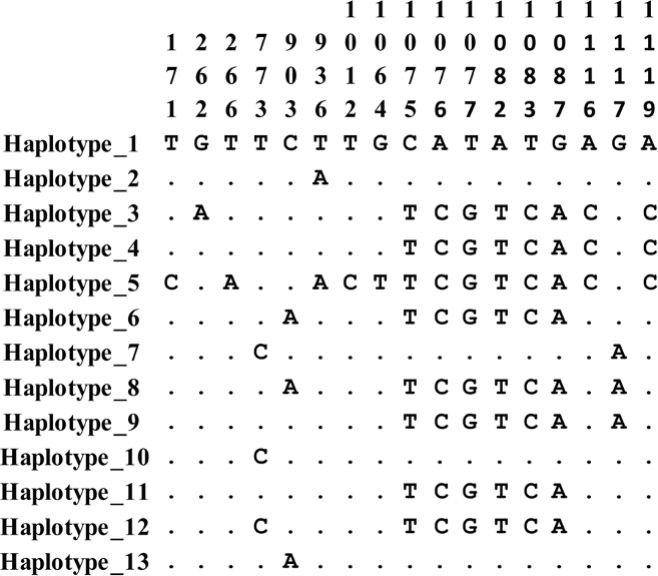
**Aligning the haplotypes found in the** ***pv41*** **gene.** The numbers in the upper part indicate the nucleotide position where a substitution was observed; the dots indicate nucleotide identity.

Table 1 gives the values for the estimators of genetic diversity. Seventeen segregant sites were observed in the sequences from different parts of the world, 12 of them being parsimony-informative sites and five singleton sites; 13 haplotypes were found (Figure 2). Aligning the proteins from *P. vivax* isolates from different geographical locations revealed substitutions in ten amino acids: N88D, E89V, A258V, Q301H, K312N, M355R, S359H, Y361F, N363D and R373G (numeration based on the Sal-I reference sequence). Ten segregant sites were found in the Colombian population (nine of them being parsimony-informative sites), giving ten haplotypes (haplotypes 1, 2, 6–13) and 0.679 ± 0.083 haplotype diversity. Haplotype 1 had 50% frequency, followed by haplotype 11 (13% frequency) and haplotype 10 (10% frequency); the remaining haplotypes had low frequency (around 3%).

**Table 1:** **Genetic diversity estimators for** ***pv41***

n	Sites	Ss	S	Ps	H	θ ^w^	*k*	π
**Worldwide diversity**								
36	1,068	17	5	12	13	0.0038 ± 0.0009	3.9	0.0037 ± 0.0006
**Local diversity**								
30	1,115	10	1	9	10	0.0023 ± 0.0007	3.1	0.0028 ± 0.0005

The estimators of genetic diversity were calculated by using the sequences obtained from the databases plus the Colombian ones (worldwide diversity) and just using those obtained in the Colombian population (local diversity).

n: number of isolates, sites: total of sites analysed (excluding gaps), Ss: number of segregant sites, S: number of singleton sites, Ps: number of parsimony-informative sites, H: number of haplotypes, *k*: average number of nucleotide differences by sequence pairs, θ^w^: Watterson estimator, π: nucleotide diversity per site.

The average number of nucleotide differences per pairs of sequences (*k*) was 3.9 when sequences from different parts of the world (worldwide diversity) were analysed and 3.1 for the Colombian population (Table 1). Low Watterson estimator (θ^w =^ 0.0038 ± 0.0009) and nucleotide diversity values (π = 0.0037 ± 0.0006) were observed when the available sequences obtained from the databases plus the Colombian ones were analysed; θ^w^ was 0.0023 ± 0.0007 and π 0.0028 ± 0.0005 for the Colombian population (Table 1). The nucleotide diversity analysis for Colombian locations showed that the Midwest was the most diverse at the *pv41* locus whilst the lowest value was found in Colombia’s South-west area (Additional file 2). The gene region having the highest π value was found between nucleotides 1,064 to 1,130.

### Evaluating the effect of natural selection on *pv41*

**Figure 3:**
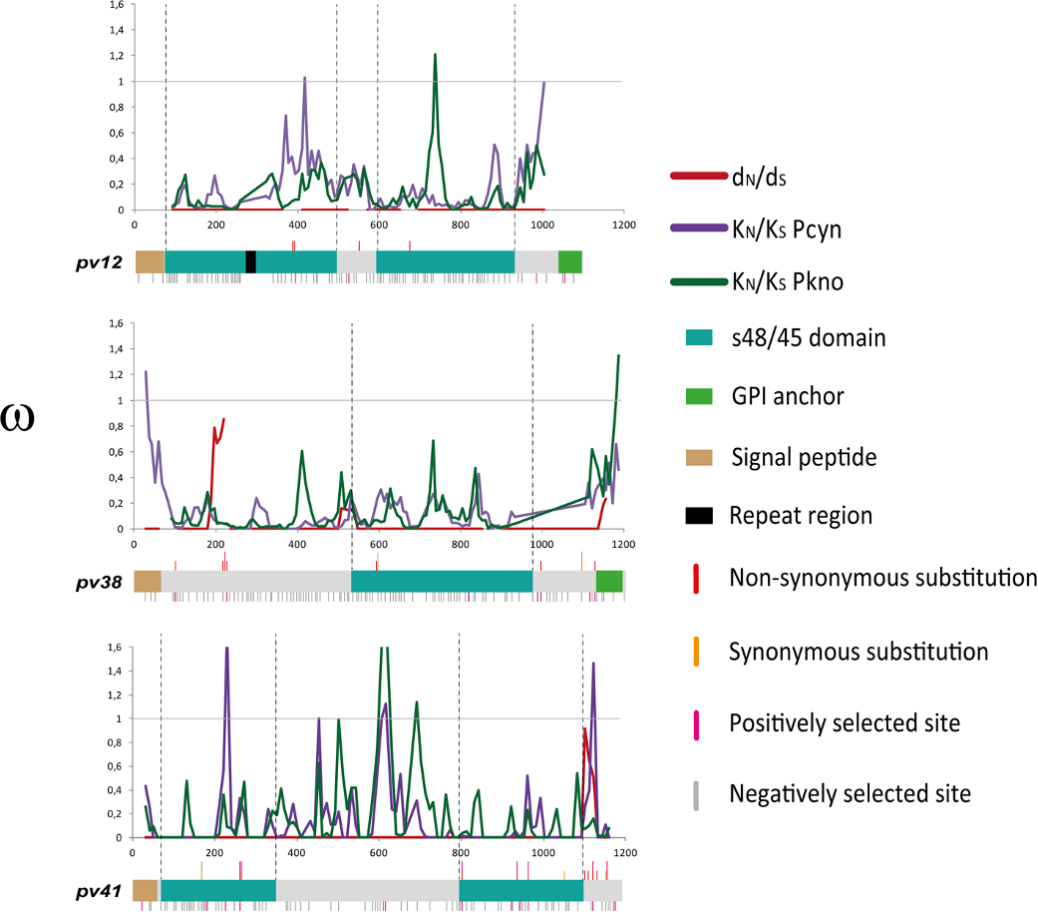
**Sliding window of omega values (ω = d**_**N**_ **/d**_**S**_ **and K**_**N**_ **/K**_**S**_ **) for three members of the 6-Cys family expressed in merozoites.** The ω values (d_N_/d_S_) for *Plasmodium vivax pv12*, *pv38* and *pv41* genes are shown in red, divergence (ω = K_N_/K_S_) between *P. vivax* and *Plasmodium cynomolgi* (Pcyn) in purple and between *P. vivax* and *Plasmodium knowlesi* (Pkno) in green. The graphical representation of each gene is given below the respective sliding window, showing the position of the segregant sites and which of them were influenced by natural selection. Intraspecies selected sites can be seen in the upper part of each gene and inter-species selected sites are indicated in the lower part. The sites were identified by the Datamonkey server. The schemes for *pv12* and *pv38* have been modified from Forero-Rodríguez *et al*. [29].

Tajima’s D, Fu and Li’s D* and F*, Fay and Wu’s H, Fu’s Fs and the K- and H-test neutrality tests did not give statistically significant values (Table 2); this meant that neutrality could not be ruled out. The differences between non-synonymous and synonymous (d_N_ - d_S_) substitutions rates throughout the gene were evaluated for estimating the effect of natural selection in *pv41*, as well as in each s48/45 domain (s48/45 N-Terminal: nucleotide 76–351 and s48/45 C-Terminal: nucleotide 784–1,095); however, no significant values were found (Table 3). The sliding window (Figure 3) for the ω (d_N_/d_S_) rate gave a ω close to 1 at the 3′-end of *pv41*, indicating a number of non-synonymous substitutions fixed within *P. vivax* in this region at a higher rate than in the rest of the sequence. Tests estimating d_N_/d_S_ for each site (codon) were then performed for identifying whether individual codons in *pv41* were under selection; seven codons were found to be under positive selection and one codon under negative selection (Figure 3). Substitutions V269A, H312Q and G384R were exclusive for the Colombian population. The K323N, H370S amino acid changes were found in Colombian isolates and some reference sequences, whilst the N88D and E89V substitutions were present in Mauritanian and South Korean sequences, respectively.

**Table 2:** **Tests based on the neutral model of molecular evolution, linkage disequilibrium and recombination for the** ***pv41*** **gene in the Colombian population**

n	Tajima	Fu and Li		Fay and Wu H	Fu Fs	K-test	H-test	Zns	ZZ	RM
	D	D*	F*							
30	0.79023	0.86738	0.9868	−1.857	−1.267	10	0.679 ± 0.08	0.3627*	0.2073*	2

*p <0.05.

**Table 3:** **Difference between the non-synonymous substitutions per non-synonymous site (d**_**N**_ **) and synonymous substitution per synonymous site (d**_**S**_ **) rate**

n	s48/45 N-terminal	s48/45 C-terminal	Complete sequences
**Worldwide isolates**	**d**_**N**_ **- d**_**S**_	**d**_**N**_ **- d**_**S**_	**d**_**N**_ **- d**_**S**_
36	−0.0001 ± 0.0008	0.0018 ± 0.0015	−0.0005 ± 0.0013
**Colombian isolate**			
30	0.0000 ± 0.0000	0.0024 ± 0.0015	0.0007 ± 0.0010

No statistically significant values were found.

The McDonald-Kreitman test was calculated for evaluating how selection had acted throughout *p41*’s evolutionary history; it revealed significant values, thereby showing that polymorphism was greater than divergence (p < 0.05) (Table 4). A sliding window for ω divergence (K_N_/K_S_, non-synonymous divergence/synonymous divergence), obtained by comparing the *P. vivax* sequences to sequences from phylogenetically close species (*P. cynomolgi* and *P. knowlesi*), gave values less than 1 in the s48/45 domains, as well as in some areas between these domains, thereby indicating that K_S_ tended to be greater than K_N_. Significant negative values (p < 0.001) were found when estimating the difference between non-synonymous and synonymous divergence (K_N_ - K_S_) (Table 5). The codon-based selection tests found 13 positively selected codons and 77 negatively selected codons at inter-species level (Figure 3).

**Table 4:** **McDonald-Kreitman test for evaluating the action of natural selection on the** ***p41*** **gene**

	*P. vivax/P. cynomolgi*			*P. vivax/P. knowlesi*		
Worldwide isolates	Fixed	Polymorphic	NI (p-values)	Fixed	Polymorphic	NI (p-values)
**Non-synonymous substitutions**	45.62	11	4.45 (0.003)	61.95	11	4.12 (0.004)
**Synonymous substitutions**	110.71	6		138.81	6	
**Colombian isolates**						
**Non-synonymous substitutions**	46.69	8	9.65 (0.000)	63.06	8	8.80 (0.001)
**Synonymous substitutions**	112.65	2		138.81	2	

The McDonald-Kreitman test involved using the sequences obtained from the databases together with the Colombian ones (worldwide isolates), and just those obtained in the Colombian population (Colombian isolates). The data regarding divergence between species was obtained by comparing *P. vivax* sequences to that from two related species: *P. cynomolgi* and *P. knowlesi*. NI: neutral index.

**Table 5:** **Difference between non-synonymous divergence per non-synonymous site (K**_**N**_ **) and synonymous divergence per synonymous site (K**_**S**_ **)**

*P. vivax/P. cynomolgi*			
n	s48/45 N-terminal	s48/45 C-terminal	Complete sequences
Worldwide isolates	K _N_ - K _S_	K _N_ - K _S_	K _N_ - K _S_
36	−0.0151 ± 0.0031*	−0.0107 ± 0.0032**	−0.0160 ± 0.0028*
**Colombian isolates**			
30	−0.0178 ± 0.0038*	- 0.0126 ± 0.0035**	−0.0174 ± 0.0030*
*P. vivax/P. knowlesi*			
**n**	**s48/45 N-terminal**	**s48/45 c-terminal**	**Complete sequences**
**Worldwide isolates**	**K**_**N**_ **- K**_**S**_	**K**_**N**_ **- K**_**S**_	**K**_**N**_ **- K**_**S**_
36	−0.0196 ± 0.0036*	−0.0107 ± 0.0034**	−0.0185 ± 0.0031*
**Colombian isolates**			
30	−0.0233 ± 0.0042*	−0.0125 ± 0.0036**	−0.0217 ± 0.0035*

K_N_ - K_S_ difference was estimated using the sequences obtained from the databases together with the Colombian ones (worldwide isolates) and just with those obtained in the Colombian population (Colombian isolates).

n: number of isolates. *p <0.000; **p <0.001.

### Linkage disequilibrium (LD) and recombination

The Z_nS_, ZZ and RM tests were calculated for determining possible associations between polymorphism and/or the presence of recombination in *pv41* (Table 2). The Z_nS_ test gave 0.3627, this being statistically significant (p < 0.05). Lineal regression between LD and nucleotide distance gave a slight reduction in LD as nucleotide distance increased, suggesting recombination events. This was confirmed when the ZZ test was calculated, giving 0.2073 (p < 0.05); two minimum recombination sites were found (Table 2). The GARD method (available from the Datamonkey web server) gave a recombination breakpoint in position 936 (number based on Sal-I sequence) confirming than intragenic recombination was involved in generating new haplotypes in *pv41*.

## Discussion

Merozoite-expressed members of the 6-Cys family in *P. falciparum* (Pf12, Pf38 and Pf41) have high RBC binding activity peptides [17], indicating that these play a role during recognition of a host cell. Previous studies have shown that members of this family are antigenic [23, 24, 27, 28] and highly conserved (*p12* and *p38*) in both *P. falciparum* and *P. vivax* [26, 29, 56, 57]. This means that they are promising candidates for inclusion in an anti-malarial vaccine, avoiding allele-specific immune responses. The *pv41* gene has been shown to be highly conserved when compared to other genes encoding antigens in *P. vivax* (e.g., *pvmsp-7* [6], *pvmsp-5* [7, 12], *pvmsp-3* [9, 10], *pvmsp-1* [5, 8]).

The *pv41* nucleotide diversity was low in the Colombian population; however, π values and haplotype number were dissimilar for each Colombian locality, suggesting different evolutionary histories possibly due to a structured population. However, this pattern could have been due to few samples having been collected from some locations. The use of neutral markers could lead to confirming whether Colombia has a structured population.

*pv41* nucleotide diversity was higher than that reported for *pv12*, but similar to that found in *pv38* [29]; however, fewer haplotypes were found in *pv41* compared to *pv38* (14 haplotypes have been reported for it in the Colombian population) [29]. Since the Pv41 protein has no membrane-anchoring domains, it could be interacting with proteins anchored to the merozoite surface. It has been shown that Pf12 and Pf41 proteins form an inverted heteroduplex on parasite membrane [25, 26]. Due to these proteins’ similarity, it is probable that Pv12 and Pv41 may also interact in *P. vivax*. This could explain the high degree of conservation found in Pv12 (π = 0.0004 ± 0.0001 [29]). If Pv41 forms a protein complex with Pv12, the latter could be masked whilst Pv41 would be more exposed to a host’s immune system, greater diversity thus being found in Pv41 (π = 0.0037 ± 0.0006) regarding Pv12 (π = 0.0004 ± 0.0001). Since such complex formation would be anti-parallel, the region most exposed to Pv41 would be the C-terminal in which high fixation of non-synonymous substitutions was observed (Figure 3).

No significant values were found in the neutrality tests based on the polymorphism frequency spectrum or the haplotype-based tests (Table 2), meaning that the hypothesis regarding neutrality could not be ruled out. Such hypothesis stated that *pv41* haplotypes could be fixed in different populations thereby producing a population structure in this locus and new *pv41* haplotypes might thus appear if new parasites populations are evaluated.

No significant values were found when the effect of natural selection was evaluated by means of the difference between non-synonymous and synonymous substitutions (d_N_ - d_S_) in either the whole gene or in each s48/45 domain (Table 3). However, the *pv41* sliding window gave a peak close to 1 at the 3′-end of the gene (Figure 3); several non-synonymous mutations would thus seem to be fixed in this region. The codon-based selection tests showed that seven out of the ten codons having mutations producing a change in the protein were positively selected (Figure 3). Three of these seven codons (V89E, H359S and G373R) produced radical substitutions (changing amino acid physical/chemical properties). The R355M substitution also produced a radical change but selection signals were not identified in this site. Such positively selected codons were predominantly found towards the gene’s 3′-end (encoding the protein’s C-terminal region) and could have been fixed to enable evading the immune system since this region would be more exposed due to the possible antiparallel formation of a Pv12/Pv41complex. Substitutions in codons 258, 301 and 312 located in the s48/45 domain could become deleterious due to them being able to alter the domain’s structure; however, they had positive selection signals. Such substitutions were conservative and maintained the amino acids’ physical-chemical characteristics, thereby enabling evasion of the immune system and maintaining the domain’s structural conformation. Interspecies ω values were higher than 1 in some regions of *p41*, mainly outside s48/45 domains. Thirteen codons were positively selected at interspecies level; amino acid fixation would allow immune evasion of the respective host. Alternatively, positive sites found in s48/45 domains (which are involved in red blood cell invasion [17]) would be a P41 adaptation to the host receptor molecule.

The Z_nS_ test had significant values, indicating LD. The linked positions were found in the 3′-end of the gene. The mutations found there led to changes in protein sequence H359S, Y361F and D363N. The first substitution (H359S) produced a radical amino acid change, which was fixed by positive selection whilst the other two changes were conservative without selection signals. Since amino acid H359S was fixed by positive selection, this led to Y361F and D363N becoming fixed due to the short physical distance between them.

Genetic diversity in *pv41* was produced by point mutations (Figure 2); however, the recombination could also have been responsible for the genetic polymorphism found in this gene. The lineal regression between LD and nucleotide distance had a slight reduction in LD as nucleotide distance increased; this may have been a consequence of recombination processes. The ZZ test gave significant values, suggesting that recombination took place in this gene. Two minimum recombination sites were found and the GARD method (available from the Datamonkey web server) identified a recombination breakpoint in position 936, meaning that recombination produced new haplotypes in *pv41*.

The McDonald-Kreitman (MK) and omega divergence tests (ω = K_N_/K_S_) were calculated for inferring natural selection signals which might have influence the evolutionary history of *p41.* The latter was calculated for the gene’s complete length and for each s48/45 domain. Significant values were found in the MK test throughout the whole gene (Table 5), polymorphism being greater than divergence; this could have resulted from weak negative selection or balancing positive selection. The latter is responsible for keeping allele variants (haplotypes) at intermediate frequencies as a mechanism for evading host immune responses; however, a major haplotype was found in the Colombian population whilst the rest occurred at low frequency. Due to the population structure reported in America [58], haplotype segregation could have led to different frequencies or new haplotypes could have diversified within American (or Colombian) subpopulations, meaning that if just one population is analysed, then balancing positive selection signals will not always be detected with population methods (Tajima, Fu and Li, Fay and Wu, Fu and K-test, and H-test). Alternatively, if balancing selection has resulted from frequency dependent selection, it would be expected that a haplotype would be presented as a major allele during a determined period of time and then become replaced by another less frequent one as an evasion mechanism. These haplotypes’ frequency must therefore be evaluated during different intervals of time in several populations involving larger sampling.

On the other hand, the ω (K_N_/K_S_) rate sliding window showed that most values obtained throughout the gene were lower than 1, indicating high synonymous substitution fixation following *P. vivax*/*P. cynomolgi*/*P. knowlesi* divergence. The same pattern was observed in *pv12* and *pv38* (Figure 3 and [29]). The difference between non-synonymous and synonymous (K_N_ - K_S_) divergence was estimated, giving significant negative values (p < 0.001) in *pv41* as well as in the s48/45 domains of this gene (Table 4). A large amount of negatively selected codons were identified which were preferentially located in the s48/45 domains (Figure 3). These results suggested that *p41* had diverged due to negative selection; such pattern was similar to that previously reported for other members of the *P. vivax* 6-Cys family [29, 56]. *pv12* and *pv38*, like *pv41*, had various codons under negative selection at interspecies level which were preferentially located in the s48/45 domains (Figure 3). Such accumulation of interspecies synonymous substitutions suggested that evolution had tried to maintain domain structure in the different members of the 6-Cys family by eliminating all deleterious mutations due to the functional importance which these domains seem to have [17, 59].

## Conclusions

6-Cys family members seem to play a role during host cell recognition [17, 59]. Due to the high degree of P12, P38 [29] and P41 protein conservation (at both intraspecies and interspecies level) given by the fixation of a large amount of synonymous substitutions, these three proteins may have evolved under strong functional constraints, possibly due to the presence of s48/45 domains which seem to have served as ligands for recognising the host cell [17, 59, 60]. Consequently, s48/45 domains should remain conserved as the resulting mutations could be deleterious; their evolution would thus have been slower regarding other functionally less important ones. Pv12, Pv38 and Pv41 thus warrant consideration as valuable candidates for developing a vaccine. However, a functional constraint does not imply that these regions may not vary. Pv41 s48/45 domains have been seen to have changes in their protein sequence, which seem to have been positively selected. Such changes conserve physical-chemical properties and thus structure/function may not become compromised, but could enable evasion of the immune response. Including Pv41 in a vaccine should thus be carefully evaluated due to the presence of variants in these regions.

This is also another aspect that must be taken into account when developing vaccines. It has been proposed that a completely effective vaccine requires the inclusion of both functional and conserved regions; however, vaccination could thus produce new selective pressure in these regions and parasites could fix mutations as an adaptation mechanism (in spite of their functional importance) and the appearance of new variants might thus reduce vaccine’s efficacy.

## Electronic supplementary material

Additional file 1: **DNA sequences from 30 isolates obtained in this study.** (TXT 34 KB)

Additional file 2: **Nucleotide diversity (π) values for subpopulations within Colombia.** n: number of isolates, Ss: number of segregant sites, S: number of singleton sites, Ps: number of parsimony-informative sites, H: number of haplotypes, *k*: average number of nucleotide differences by sequence pairs, π: nucleotide diversity per site. (PDF 113 KB)
