# Supplementary material for: Low genetic diversity in the locus encoding the Plasmodium vivax P41 protein in Colombia’s parasite population
Source: Malar J. 2014 Sep 30;13:388. doi: 10.1186/1475-2875-13-388 (PMC4190493; doi:10.1186/1475-2875-13-388)
Supplement: Supplementary file 2 — Additional file 2: Nucleotide diversity (π) values for subpopulations within Colombia. n: number of isolates, Ss: number of segregant sites, S: number of singleton sites, Ps: number of parsimony-informative sites, H: number of haplotypes, k: average number of nucleotide differences by sequence pairs, π: nucleotide diversity per site. (PDF 113 KB) [file 12936_2014_3548_MOESM2_ESM.pdf]

Additional file 2 Nucleotide diversity (π) values for subpopulations within Colombia

n	Location	Ss	S	Ps	H	*k*	π
14	North-west	9	1	8	6	3.6	0.0032 (0.0005)
3	Midwest	7	7	0	2	4.7	0,0042 (0.0019)
4	South-west	1	1	0	2	0.5	0.0004 (0.0002)
9	South-east	9	1	8	4	3.3	0.0029 (0.0011)

n: number of isolates, Ss: number of segregant sites, S: number of singleton sites, Ps: number of parsimony-informative sites, H: number of haplotypes, *k*: average number of nucleotide differences by sequence pairs, π: nucleotide diversity per site.
